# The Inhibitory Effect of 6-Gingerol on Ubiquitin-Specific Peptidase 14 Enhances Autophagy-Dependent Ferroptosis and Anti-Tumor *in vivo* and *in vitro*

**DOI:** 10.3389/fphar.2020.598555

**Published:** 2020-11-13

**Authors:** Yun Tsai, Changbo Xia, Zhongwen Sun

**Affiliations:** ^1^ Nanjing University of Chinese Medicine, Nanjing, China; ^2^ Shandong University of Traditional Chinese Medicine, Jinan, China; ^3^ Taishan Hospital of Shandong Province, Tai’an, China

**Keywords:** 6-gingerol, autophagy, ferroptosis, A549 cell, ubiquitin-specific peptidase 14

## Abstract

Lung cancer is the most common malignant tumor and is the leading cause of cancer-related deaths worldwide. Extraction of bioactive substances from herbs is considered as an alternative method to traditional treatment. 6-Gingerol is a naturally occurring phenol found in ginger that can be used to treat tumors and suppress inflammation. To determine whether 6-Gingerol can be used as a therapeutic agent for tumors. In this study, tumor-bearing mice were used as an animal model and A549 as a cell model. Western blot was used to detect the expression of autophagy related proteins ubiquitin-specific peptidase 14 (USP14), Beclin1, microtubule-associated protein light chain 3 (LC3) and ferroptosis related proteins nuclear receptor coactivator 4 (NCOA4), ferritin heavy chain 1 (FTH1), transferrin receptor 1 (TfR1), glutathione peroxidase 4 (GPX4), activating transcription factor4 (ATF4) *in vivo* and *in vitro*. MTT and EdU were used to detect the viability of A549 cells. H&E and immunofluorescence were used to localize and detect the expression of proteins. The detection of reactive oxygen species was performed using fluorescence probes. It was found that the administration of 6-Gingerol decreased the expression of USP14, greatly increased the number of autophagosomes, reactive oxygen species (ROS) and iron concentration, decreased the survival and proliferation rate of A549 cells, and significantly decreased tumor volume and weight. The results indicate that 6-Gingerol inhibits lung cancer cell growth via suppression of USP14 expression and its downstream regulation of autophagy-dependent ferroptosis, revealing the function and efficacy of 6-Gingerol as a therapeutic compound in A549 and its possible mechanism of action.

## Introduction

Lung cancer has the highest incidence of all cancers, with a mortality rate accounts for 18.4% of cancer related death (Siegel et al., 2017; Bray et al., 2018). Currently, traditional chemotherapy, radiation therapy, targeted therapy and surgery are the main clinical strategies for the treatment of lung cancer. However, in most cases, the clinical effects are not satisfactory (Maas et al., 2010). Compared to these therapies, natural products usually have multiple benefits with minimal side effects, which makes them suitable for cancer treatment (Shanmugam et al., 2017). Therefore, the discovery of new anti-lung cancer drugs is necessary for the treatment of lung cancer.

Cell death occurs in multicellular organisms and includes necrosis, apoptosis and autophagy to maintain tissue function and morphology (Majno and Joris, 1995). The autophagy process has an important inhibitory effect on tumor survival and proliferation (Wei et al., 2008). Beclin1 is an essential molecule in the formation of autophagosomes and is also a tumor suppressor (Yue et al., 2003). Expression levels of Beclin1 tend to increase during autophagy. Ubiquitin-specific protease (USP) is a cysteine protease, and both USP10 and USP13 mediate the deubiquitination activity of Beclin1 to control its protein stability (Liu et al., 2011). USP19 is a deubiquitinase localized to the endoplasmic reticulum that stabilizes Beclin1 for deubiquitination (Tripathi et al., 2014). The deubiquitination of USP14 inhibits the autophagic occurrence, and USP14 expression is increased in a variety of cancers, these studies make USP14 a potential approach to cancer treatment (Xu et al., 2016).

It has shown that autophagy can regulate ferroptosis by degrading ferritin (Hou et al., 2016), and ferritin can promote ferroptosis (Mancias et al., 2014). Ferroptosis is a new form of cell death triggered by lipid peroxidation in an iron-dependent manner (Angeli et al., 2017). Ferroptosis has been classified as one of the regulated cell death (Galluzzi et al., 2018), and the immune system may prevent tumorigenesis in part through ferroptosis (Wang et al., 2019). The biomarkers lipid peroxidation and lipid reactive oxygen species (ROS) can be used to identify the occurrence of ferroptosis (Li et al., 2017).

6-Gingerol is a naturally occurring phenol in ginger (*Zingiber officinale Roscoe*), that has been shown to have anti-inflammatory, anti-tumor and antioxidant bioactivities (Koch et al., 2017; Zhang et al., 2017; Alencar et al., 2018). 6-Gingerol showed an anti-proliferative effect on cervical cancer cells (HeLa, CaSki, SiHa) *in vitro* (Rastogi et al., 2015) and induced TRAIL-mediated apoptosis in glioblastoma (U87) tumor cell lines (Lee et al., 2014). 6-Gingerol attenuated colorectal cancer via anti-inflammatory, anti-proliferative and apoptotic mechanisms in mice (Farombi et al., 2020). During treatment with 6-Gingerol, the content of ROS in tumors increased, leading to the inhibition of growth and induction of apoptosis.

The present research aimed to determine whether ferroptosis is related to tumor death and to reveal its underlying mechanism. We hypothesized that 6-Gingerol promotes rust disease and leads to tumor death by modulating USP14 expression and inducing Beclin 1-dependent autophagy.

## Materials and Methods

### Reagents

For *in vivo* and *in vitro* experiment, 6-Gingerol (purity ≥ 98%) from Aladdin Ltd (Shanghai, China) was dissolved in 50% DMSO and diluted with double distilled water, the final concentrations of DMSO were less than 0.1% to reduce cytotoxicity (Liu et al., 2020).

### Cells and Animals Experiments

Cancer cell line (A549, Solarbio, China) originating from human lung tumors were cultured in RPMI-1640 medium (Gibco, CA) with 10% fetal bovine serum (FBS; Biological Industries, Israel) and antibiotics (100 μg/ml penicillin -streptomycin, Beyotime, China). The cell cultures were incubated in an environment containing 5% CO_2_ at 37°C. Cells were divided into four groups: the Con group (control, no treatment), 20-Gin group (20 μM 6-Gingerol), 40-Gin group (40 μM 6-Gingerol) and 80-Gin group (80 μM 6-Gingerol). The recombinant lentivirus vectors for USP14 was provided by Genechem (Shanghai, China) for subsequent USP14-OE experiments.

BALB/cNude (6–8 weeks of age) mice were purchased from Hangzhou Ziyuan Experimental Animal Technology Co. Ltd. (SYXK-20180049) for this study. The mice were housed under specific pathogen-free conditions at 23°C and given free access to food and water. The left flank of mice was subcutaneously inoculated with A549 tumor-cell suspension (5 × 10^6^ cells/100 μL) to prepare A549 tumor xenografts (Zhang et al., 2016). Three days after tumor cell inoculation, the mice were divided into three groups (*n* = 8): Con group (control group, no treatment), L-Gin group (0.25 mg/kg/day 6-Gingerol), H-Gin group (0.5 mg/kg/day 6-Gingerol), which were administered orally daily until the end of the experiments. Mice were killed when their minor axis of tumors were longer than 20 mm. All experiments with mice were approved by ethics committee.

### Tumor Detection in Nude Mice

Tumor diameter was measured every 2 days and tumor volume (*V*; mm^3^) was calculated using Eq. 1. After the mice were killed, the tumors were removed and weighed.

V=major axis×minor axis×height×0.52 (1)

### Determination of Iron Content, Malondialdehyde and Superoxide Dismutase

Total levels of iron in different groups were analyzed using the Iron Assay kit (ab83366, Abcam, United Kingdom). Tissues homogenates/cells were lyzed in four volume of iron assay buffer and centrifuge at 16,000 × *g* for 10 min to remove insoluble materials. Total iron (Fe^3+^ plus Fe^2+^) was determined by adding 5 µL of iron reducer were added to 50 µL of sample, and 100 μL iron probe solution was added into samples and incubated in the dark at 25°C for 60 min. Absorbance was measured at 593 nm wavelength using a micro spectrophotometer (Nanodrop, Thermo).

The content of MDA was measured using Malondialdehyde (MDA) content detection kit (BC0025, Solarbio, China) and the absorbance of the supernatant was determined at 532 nm. The superoxide dismutase (SOD) activity detection kit (BC0175, Solarbio, China) was used to measure the absorbance at 425 nm to detect the SOD activity in the sample.

### Pathomorphological Detection

After being fixed in 4% paraformaldehyde for 24 h, each tumor was embedded in paraffin and cut into 3 mm using a microtome (Histocore Biocut, Thermo Fisher Scientific, USA). Hematoxylin and eosin (H&E) method was used to stain the paraffin sections prepared by slicer (Histocore Biocut, Thermo Fisher Scientific, America), and then the sections were dehydrated twice. The sections were sealed with glass and the morphology of the cells was observed under a microscope (Olympus, Japan).

For immunostaining, tumors were incubated with the ROS fluorescent probe dihydroethidium (DHE, No. D1008; Us). DHE was oxidized by ROS to ethidine oxide, which can be mixed with DNA to produce red fluorescence. Red fluorescence was observed with a fluorescence microscope (BX63, Olympus, Japan). To detect ROS in the cells, cells were subjected to a ROS assay using the DCFA-DA reactive oxygen ROS fluorescent probe (D6470, Solarbio, China) according to the manufacturer’s instructions.

### Cell Viability and Proliferation Activity Detection

The cells were spread in a 96-well plate with 5,000 cells per well. After 24 h of adhesion culture, the cells were treated with 20, 40, 80 μM 6-Gingerol. After further culture for 48 h, the cell viability rate was determined by MTT method. The viability of A549 cell was measured by MTT (M1020, Solarbio, China) according to the manufacturer'|’s instructions.

The EdU apollo 567 *in vitro* kit (CA1170, Solarbio, China) was used to measure cell proliferation. The cells were spread in a 48-well plate with 5,000 cells per well. After 24 h of adhesion culture, 20, 40, 80 μM 6-Gingerol were given. After continued incubation for 48 h, the complete medium containing EdU was replaced and incubated for 2 h, and fixed staining was conducted according to the EdU kit instructions. The EdU-positive rate was recorded according to merged pictures of EdU and DAPI.

### Detection of Autophagy

The cells were spread in 24-well plates with 4 × 10^4^ cells/well. After 24 h of adhesion culture, ad-green fluorescent protein (GFP)-microtubule-associated protein light chain 3 (LC3B) (C3006, Beyotime, China) adenovirus transfection was conducted. After 24 h of transfection, the culture containing the corresponding concentration of 6-Gingerol in each group was replaced. After 48 h of culture, the cells were fixed and stained with DAPI for photographing.

### Western Blot Assay

The tumor and A549 cell lysates were prepared, washed with cold PBS, resuspended in a lysis buffer and sonicated the lysate. The proteins were separated on 10–15% SDS gels and transferred to nitrocellulose membranes. After incubation with 1:1,000 primary antibody dilution buffer for 1 h, Goat Anti-Mouse IgG (H + L) HRP (1:5,000, No. S0002, Affinity) or Goat Anti-Rabbit IgG (H + L) HRP (1:5,000, No. S0001, Affinity) were used as secondary antibodies and developed by enhanced chemiluminescence. The antibodies used for immunoblotting were ubiquitin-specific peptidase 14 (USP14, 1191S, CST, USA), Beclin 1 (3,738, CST, USA), nuclear receptor coactivator 4 (NCOA4, ab86707, Abcam, United Kingdom), microtubule-associated protein light chain 3 (LC3 I and LC3 II 12741, CST, USA), ferritin heavy chain 1 (FTH1, 3,998, CST, USA), transferrin receptor 1 (TfR1, ab1086, Abcam, United Kingdom), glutathione peroxidase 4 (GPX4, ab125066, Abcam, United Kingdom), activating transcription factor4 (ATF4, ab184909, Abcam, United Kingdom) and β-tubulin (2146S, CST, USA).

### Co-Immunoprecipitation (Co-IP) and Ubiquitylation Assay

Co-IP was conducted following the methods described previously (Dixit et al., 2014). 1 μg of Beclin 1 (3,738, CST, USA) antibody was added to the lysis buffer and incubated overnight at 4°C. 10 μL of Protein A/G Plus agarose beads were added to the lysate buffer and incubated with slow shaking for 2 h at 4°C. At the end of the immunoprecipitation reaction, the supernatant was removed by centrifugation at 3,000 rpm for 3 min at 4°C. Precooling PBS was used to wash the precipitate for several times. 2 × loading buffer was added to the precipitate and denatured for 5 min at 95°C. The supernatant was subjected to western blot with a K63-linked specific polyubiquitin (12,930, CST, USA) antibody to detect the target protein.

### Statistical Analysis

The unpaired *t*-test were used for comparison between two groups. All data are presented as mean ± standard deviation (mean ± SD) of three independent experiments. For multiple comparison, the one-way ANOVA followed by the post-hoc test was used. All statistical analysis was performed using GraphPad Prism 7.0 software. Results were considered to be statistically significant for values *p < 0.05, **p < 0.01.

## Results

### 6-Gingerol Suppresses Tumor Growth in Tumor

**FIGURE 1:**
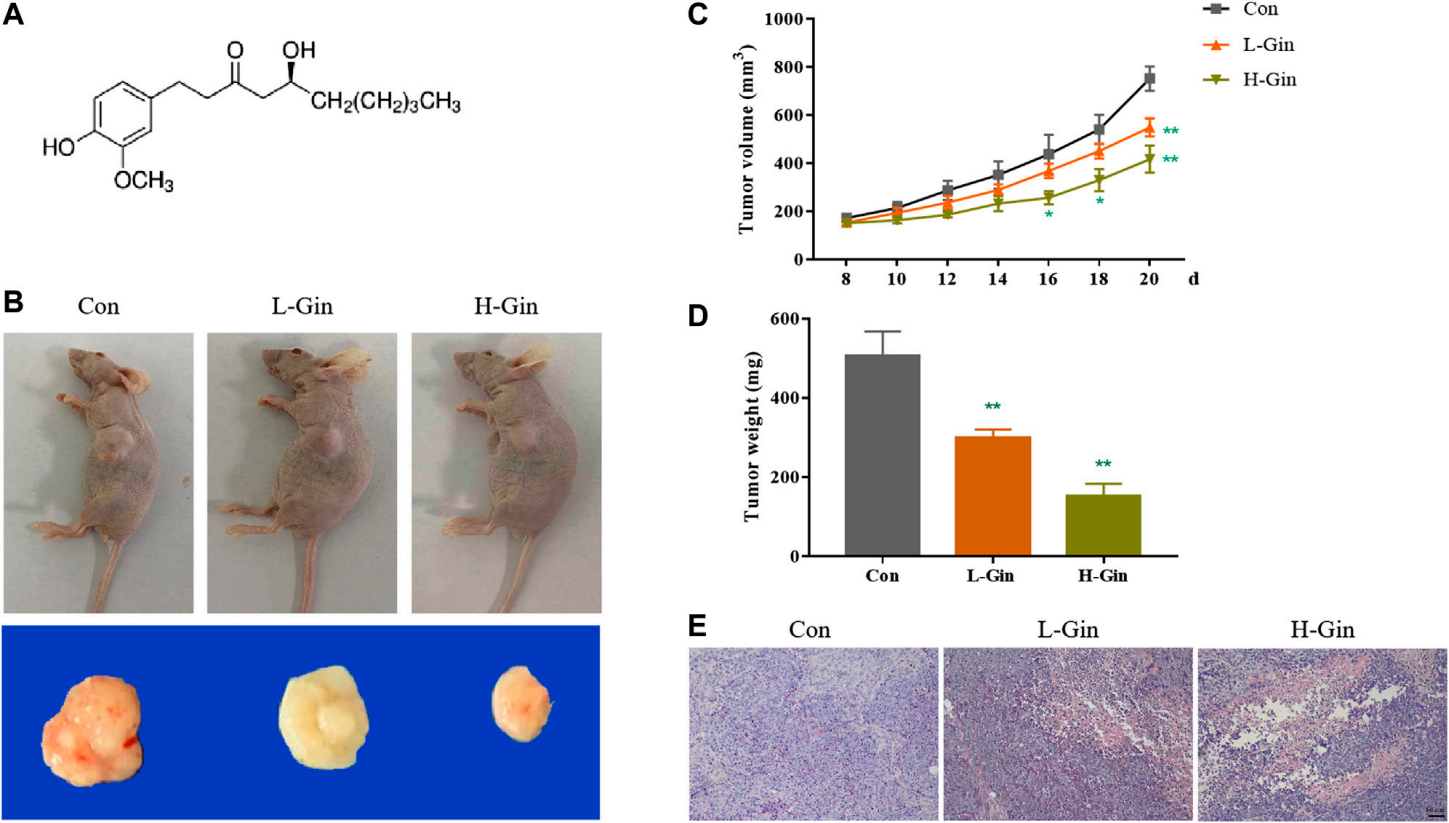
6-Gingerol suppresses growth of tumor. **(A)** Chemical structure of 6-Gingerol; **(B)** The pictures of tumor masses in nude mice; **(C)** 6-Gingerol inhibited the increase of tumor volume in nude mice; **(D)** 6-Gingerol inhibited the increase of tumor weight in nude mice; **(E)** The histological features of tumor tissues. Con: control, L-Gin: 0.25 mg/kg/day 6-Gingerol, H-Gin: 0.5 mg/kg/day 6-Gingerol. Compared with the control group, *p < 0.05, **p < 0.01. Data are expressed as mean ± SD (*n* = 8).

The chemical structure of 6-Gingerol was showed in Figure 1A. Figure 1B showed a picture of the tumor mass collected on the 20th day. The L-Gin and H-Gin group had effective and dose-dependent inhibition of tumor growth, and the H-Gin group had greater inhibition than the of L-Gin group (Figures 1C,D). As seen in Figure 1E, 6-Gingerol treatment of tumor-bearing mice caused massive infiltration of cells in the tumor. The above results demonstrate that 6-Gingerol had great anti-tumor activity *in vivo*.

### 6-Gingerol Enhances the Accumulation of Reactive Oxygen Species and Iron in Tumor

**FIGURE 2:**
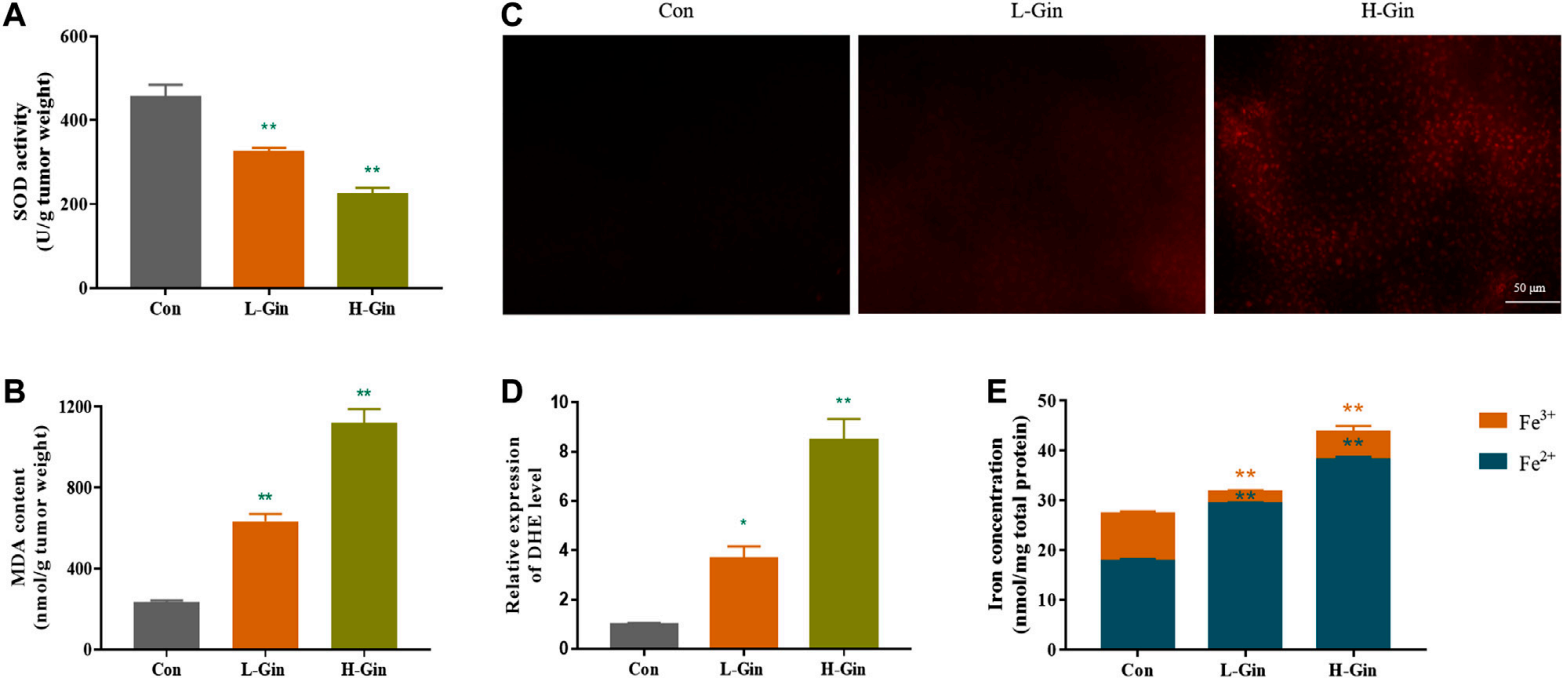
6-Gingerol enhances the accumulation of ROS and iron in tumor. **(A)** The activity of SOD in tumor tissues; **(B)** The content of MDA in tumor tissues; **(C)** The ROS fluorescence (DHE) of tumor tissues; **(D)** The histogram of ROS content of tumor tissues; **(E)** The iron concentration in tumor tissues. Con: control, L-Gin: 0.25 mg/kg/day 6-Gingerol, H-Gin: 0.5 mg/kg/day 6-Gingerol. Compared with the control group,*p < 0.05, **p < 0.01. Data are expressed as mean ± SD (*n* = 3).

Since lipid peroxidation and iron accumulation are features of ferroptosis (Stockwell et al., 2017), we next measured the iron content and lipid peroxidation levels of tumor tissues. In Figure 2A, the SOD activity of the L-Gin and H-Gin groups was significantly lower than that of the control group (*p* < 0.05). On the contrary, the MDA contents in the L-Gin and H-Gin groups were observably higher than that in the control group (*p* < 0.05, Figure 2B). The brightness of ROS-DHE probe in the L-GIN and H-GIN groups was remarkably higher than that in the control group (*p* < 0.05, Figures 2C,D). Compared with the control group, there was a significant accumulation of Fe^2+^ in the tumor tissue of 6-Gingerol treatment of tumor-bearing mice (Figure 2E).

### 6-Gingerol Inhibits the Growth of A549 Cells

**FIGURE 3:**
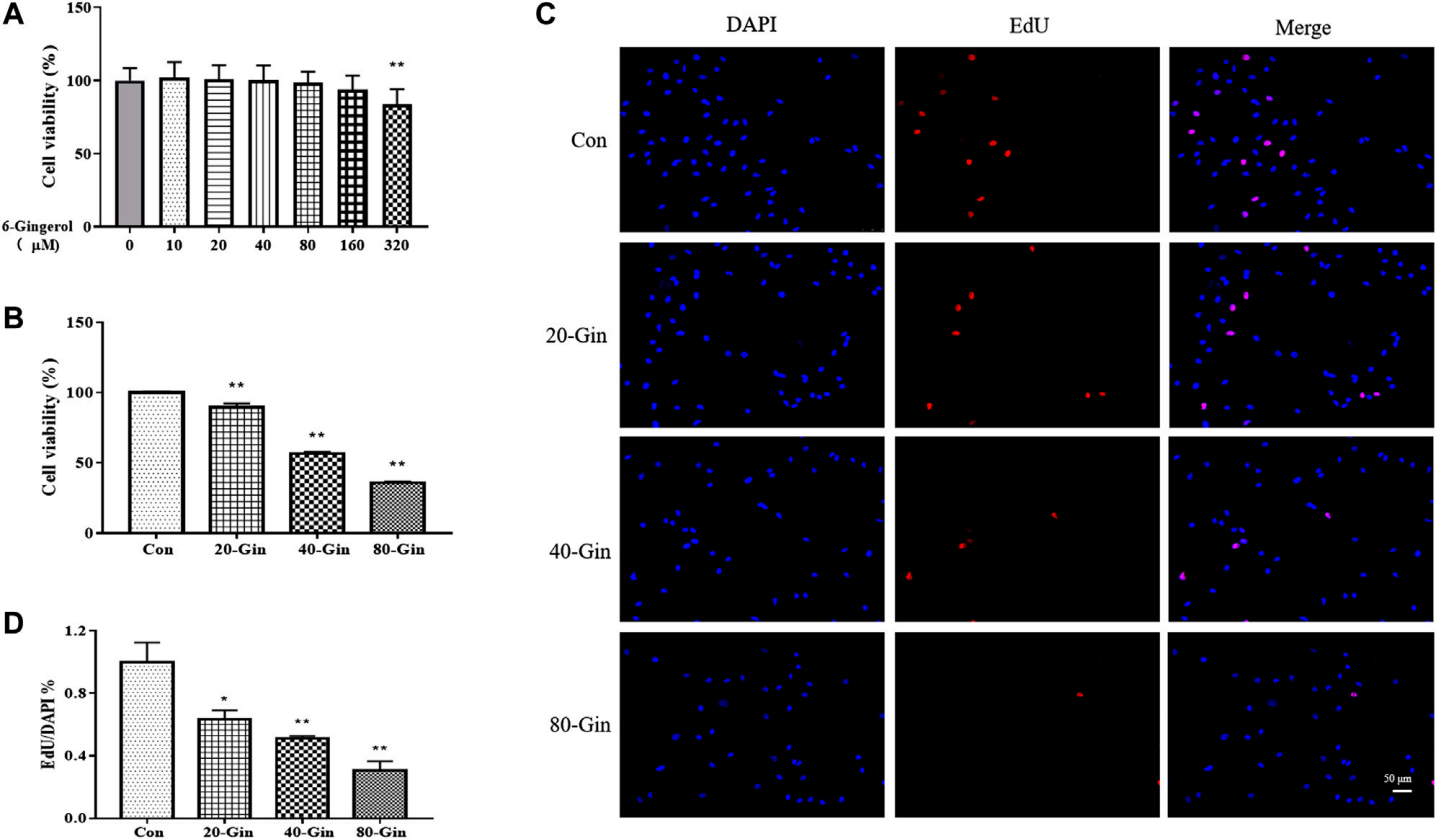
6-Gingerol inhibits the growth of A549 cells. **(A)** The viability of CCD19-Lu cells with 6-Gingerol at different concentrations (0–320 μM); **(B)** The viability of A549 cells with 6-Gingerol at different concentrations; **(C)** The representative images of EdU and DAPI; **(D)** The quantitative positive rate of EdU. Con: control, 20-Gin: 20 μM 6-Gingerol, 40-Gin: 40 μM 6-Gingerol, 80-Gin: 80 μM 6-Gingerol. Compared with the control group, *p < 0.05, **p < 0.01. Data are expressed as mean ± SD (*n* = 3).

Before studying the effect of 6-Gingerol treatment on the viability of A549 cells, the effect of different concentrations (0–320 μM) of 6-Gingerol on the viability of CCD19-Lu cells was determined. 6-Gingerol at concentrations from 0 to 160 μM did not significantly activity inhibit the viability of on CCD19-Lu cells (Figure 3A). Figure 3B showed that the viability of A549 cells were significantly inhibited by 6-Gingerol (*p* < 0.05). EdU analysis was used to evaluate the proliferative capacity of A549 cells treated with different concentrations (20–80 μM) of 6-Gingerol. The EdU incorporation test showed that 6-Gingerol reduced the positive rate of EdU in A549 cells, indicating that 6-Gingerol could inhibit the proliferative activity of A549 cells (Figures 3C,D).

### 6-Gingerol Exacerbates Autophagy and Enhances the Accumulation of Reactive Oxygen Species and Iron in A549 Cells

**FIGURE 4:**
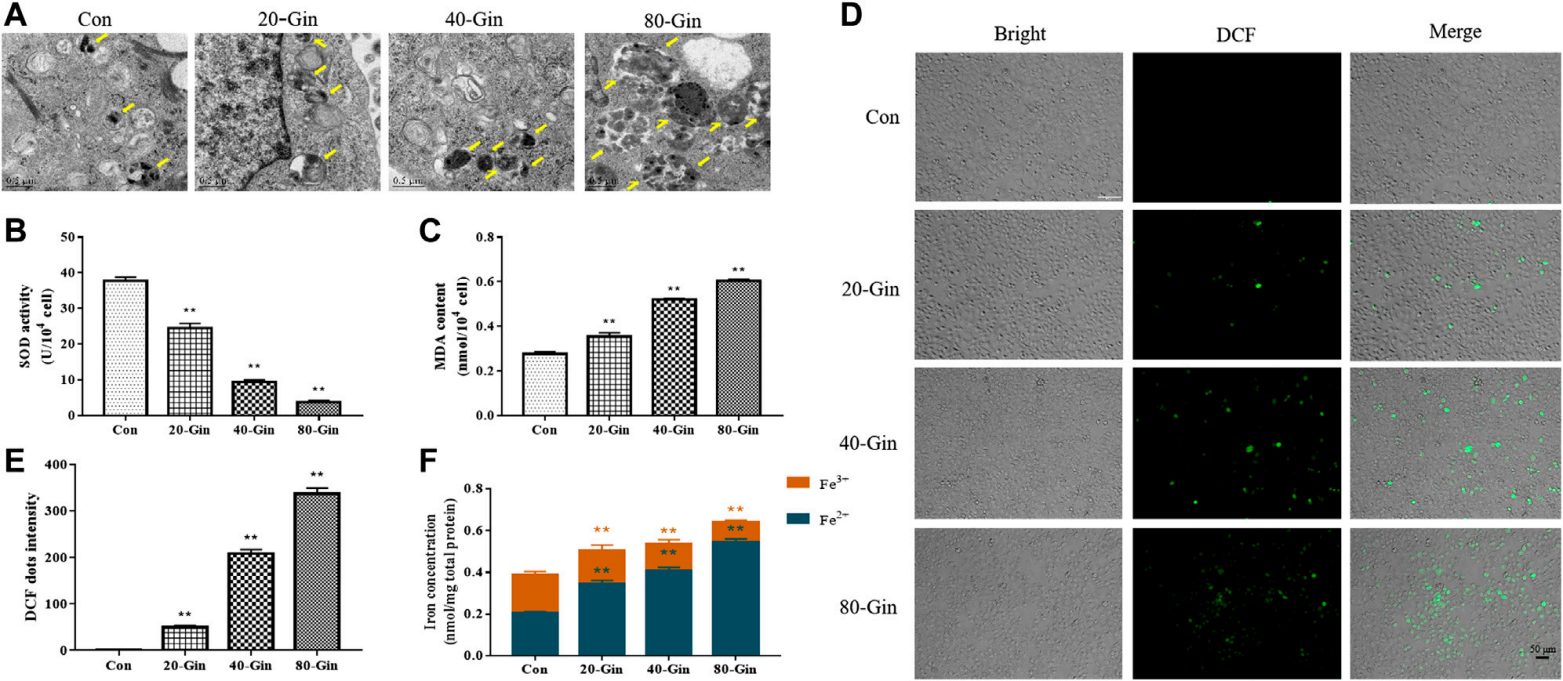
6-Gingerol exacerbates autophagy and enhances the accumulation of ROS and iron in A549 cells. **(A)** The autophagy electron microscope image of A549 cells, and yellow arrows marked the autophagy structure; **(B)** The activity of SOD in A549 cells; **(C)** The content of MDA in A549 cells; **(D)** The ROS fluorescence (DCF) of A549 cells; **(E)** The histogram of ROS content of A549 cells; **(F)** The iron concentration in A549 cells. Con: control, 20-Gin: 20 μM 6-Gingerol, 40-Gin: 40 μM 6-Gingerol, 80-Gin: 80 μM 6-Gingerol. Compared with the control group, *p < 0.05, **p < 0.01. Data are expressed as mean ± SD (*n* = 3).

From the TEM image in Figure 4A, it could be found that more autophagosomes were observed in the A549 cells treated with 6-Gingerol than that in the control group, which may be due to the up-regulation of autophagy by 6-Gingerol. In Figure 4B, the SOD activity of the 20-Gin, 40-Gin and 80-Gin groups was significantly lower than that of the control group (*p* < 0.05). With the decrease of SOD activity, the MDA content in the 20-Gin, 40-Gin and 80-Gin groups was higher than that in the control group observably (*p* < 0.05, Figure 4C). The brightness of ROS-DCF probe in the 20-Gin, 40-Gin and 80-Gin groups was significantly higher than that in the control group (Figures 4D,E, *p* < 0.05). In addition, TfR1 has been regarded as a marker of malignant phenotype for tumor (Li et al., 2010), promoting the entry of Fe^3+^ into cells to become Fe^2+^ for the Fenton reaction. As shown in Figure 4E, as the concentration of 6-Gingerol increased, the intracellular Fe^2+^ content increased. The above results indicated that 6-Gingerol could promote autophagy and increase the accumulation of intracellular ROS and Fe^2+^ content.

### 6-Gingerol Regulates the Expression of Autophagy and Ferroptosis Related Proteins *in vivo* and *in vitro*

**FIGURE 5:**
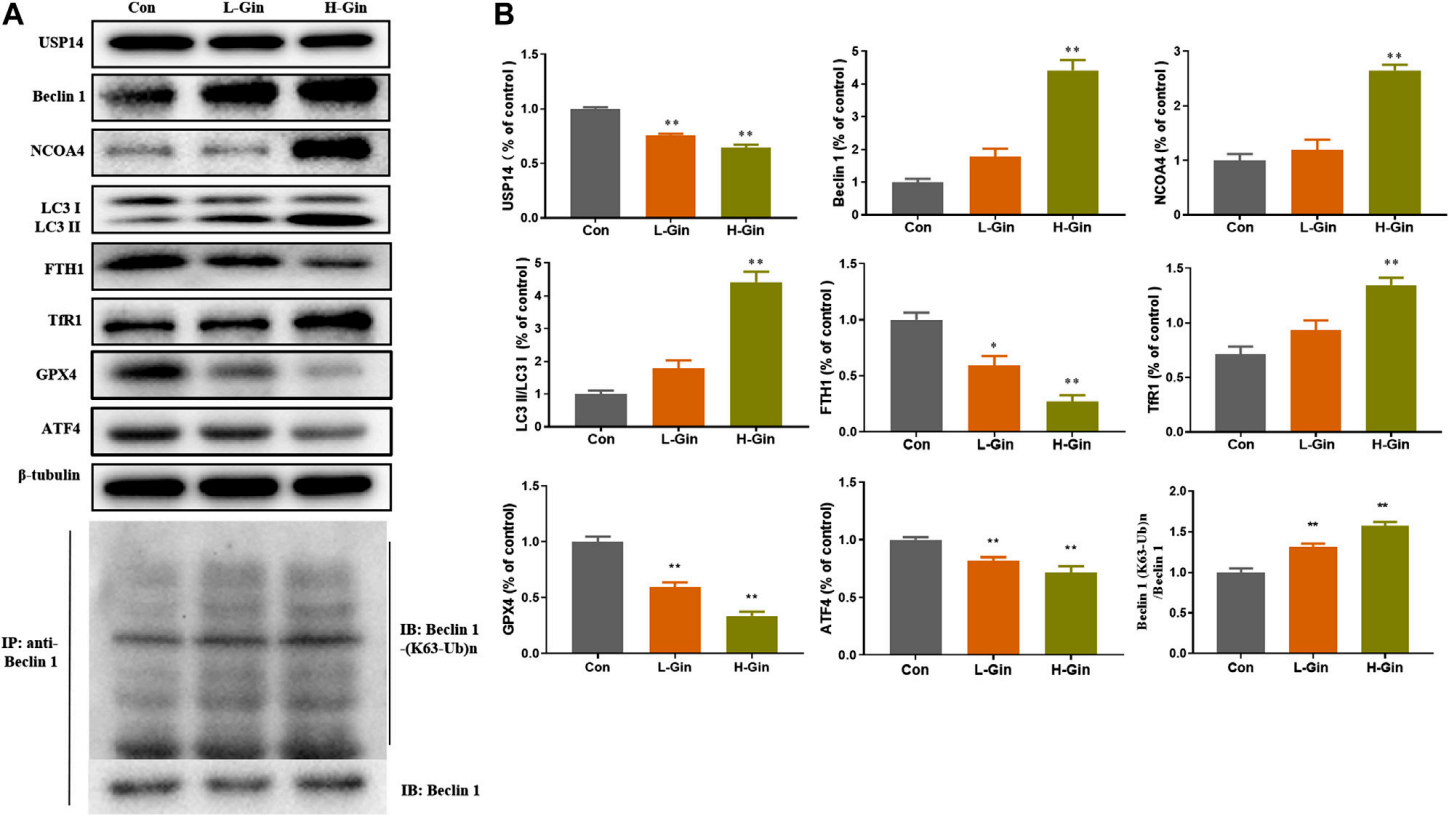
6-Gingerol regulates the expression of autophagy and ferroptosis related proteins in tumor. **(A)** The western blot analysis of USP14, Beclin 1, NCOA4, LC3 I, LC3 II, FTH1, TfR1, GPX4 and ATF4, Co-IP analysis of ubiquitination of K63 on Beclin one; **(B)** The densitometric analysis of the bands was presented as the relative ratio of USP14, Beclin 1, NCOA4, LC3 II/LC3 I, FTH1, TfR1, K63 ubiquitination on Beclin one to Beclin 1. Con: control, L-Gin: 0.25 mg/kg/day 6-Gingerol, H-Gin: 0.5 mg/kg/day 6-Gingerol. Compared with the control group, *p < 0.05, **p < 0.01. Data are expressed as mean ± SD (*n* = 3).

**FIGURE 6:**
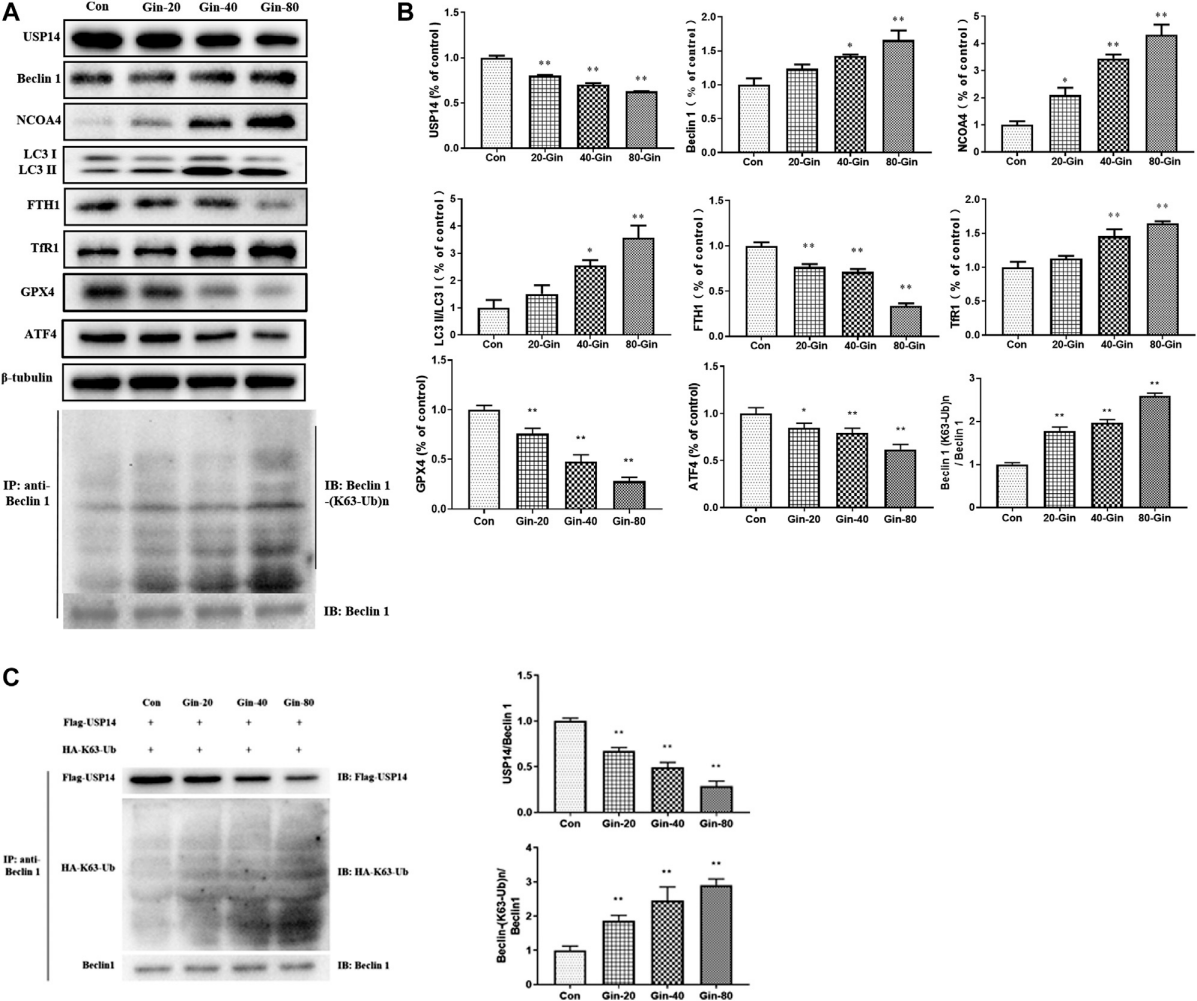
6-Gingerol regulates the expression of autophagy and ferroptosis related proteins in A549 cells. **(A)** The western blot analysis of USP14, Beclin 1, NCOA4, LC3 I, LC3 II, FTH1, TfR1, GPX4 and ATF4, Co-IP analysis of ubiquitination of K63 on Beclin one; **(B)** The densitometric analysis of the bands was presented as the relative ratio of USP14, Beclin 1, NCOA4, LC3 II/LC3 I, FTH1, TfR1, K63 ubiquitination on Beclin one to Beclin 1. **(C)** *In vitro* K63-linked deubiquitination of Beclin one by USP14. Con: control, 20-Gin: 20 μM 6-Gingerol, 40-Gin: 40 μM 6-Gingerol, 80-Gin: 80 μM 6-Gingerol. Compared with the control group, *p < 0.05, **p < 0.01. Data are expressed as mean ± SD (*n* = 3).

In view of the aforementioned autophagy phenomenon and changes in iron concentration, western blot was performed to detect the related proteins. Moreover, we confirmed that 6-Gingerol could increase the expressions of Beclin-1, LC3 I, LC3 II, NCOA4 and TfR1, and down-regulate the expressions of USP14, FTH1, GPX4 and ATF4 *in vivo* (Figures 5A,B). Similar to the *in vivo* results, 6-Gingerol could increase the expression of Beclin-1, LC3 I, LC3 II, NCOA4 and TfR1, and down-regulate the expression of USP14, FTH1, GPX4 and ATF4 (Figures 6A,B) *in vitro*. As shown in Figure 6C, 6-Gingerol promoted autophagy by inhibiting the deubiquitination of the K63 site of beclin1 by USP14. The above results suggested that 6-Gingerol inhibited the expression of USP14 and FTH1 and up-regulated the expression of other proteins *in vivo* and *in vitro*.

### 6-Gingerol Up-Regulates the Expression of Light Chain 3 and Ferritin Heavy Chain 1 in USP14-OE Cells

**FIGURE 7:**
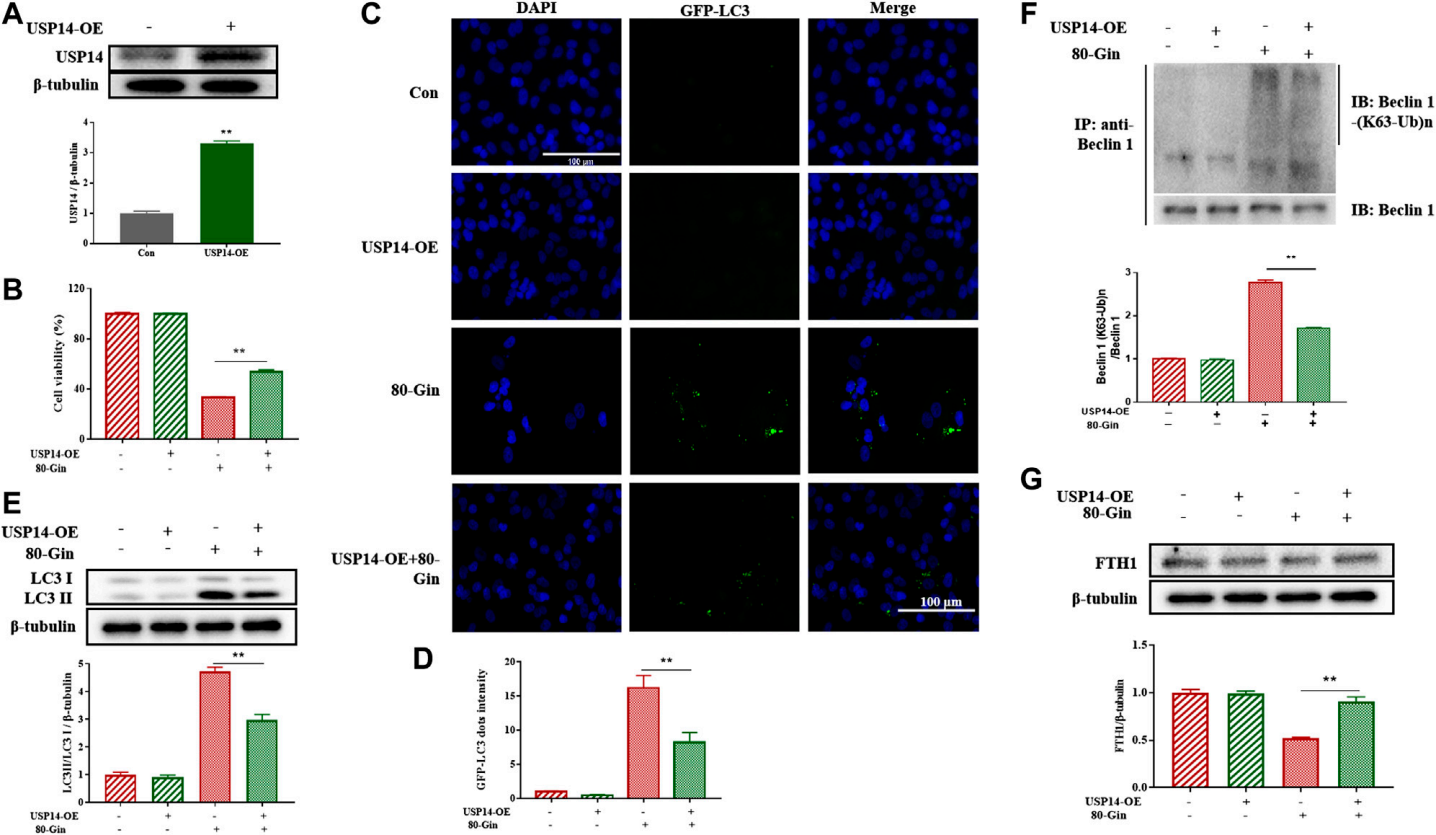
6-Gingerol up-regulates the expression of LC3 and FTH1 in USP14-OE cells. **(A)** The western blot analysis of USP14 overexpression; **(B)** The cell viability of USP14-OE cells treated with 6-Gingerol; **(C)** The representative images of GFP-LC3 and DAPI; **(D)** The quantitative positive rate of GFP-LC3; **(E)** The western blot analysis of FTH1 in USP14-OE cells; **(F)** The western blot analysis of LC3 in USP14-OE cells; **(G)** The Co-IP analysis of ubiquitination of K63 on Beclin one in USP14-OE cells. Data are expressed as mean ± SD, *p < 0.05, **p < 0.01 (*n* = 3).

To further investigate the role of USP14 on autophagy in A549 cells, we overexpressed USP14 in cells. USP14 was successfully overexpressed in cells (Figure 7A), and the expression of USP14 in the USP14-OE treated with 6-Gingerol group was lower than that in the control group (Figure 7B). The LC3 protein is involved in the formation of autophagosomes, so it is usually characterized as an autophagy marker (Rao et al., 2019). In Figures 7C,D, after 6-Gingerol treatment, the GFP fluorescence brightness of the 80-Gin group was significantly higher than that of the untreated control group, whereas the USP14-OE + 80-Gin group was significantly lower than the 80-Gin group (*p* < 0.05). Since USP14 could regulate autophagy in a Beclin 1-dependent manner (Lee et al., 2016), we futher investigated whether six gingerol regulates autophagy through Beclin1 de-ubiquitylation of K63 ubiquitin. The expressions of LC3II/LC3I and K63 ubiquitination of Beclin one in USP14-OE + 80-Gin group were higher than that in 80-Gin group, and expression of FTH1 in 80-Gin group was significantly lower than that in USP14-OE + 80-Gin group (*p* < 0.05, Figures 7E,F). These results all demonstrated 6-Gingerol could promote autophagy and ferroptosis by inhibiting the deubiquitination of the K63 site of USP14-mediated Beclin1 ubiquitination.

## Discussion

Lung cancer is the most common malignancy and the leading cause of cancer-related deaths worldwide. Lung cancer is mainly treated with Cisplatin, Permetrexide and erlotinib (Li et al., 2018), but the clinical application of these drug is limited due to their toxicity and resistance, so new anti-lung cancer drugs are urgently needed. 6-Gingerol is a phenolic substance naturally present in ginger (*Zingiber officinale Roscoe*) that has been shown to have anti-inflammatory, anti-tumor and antioxidant biological activities (Koch et al., 2017; Zhang et al., 2017; Alencar et al., 2018). Therefore, in the study, the effects and possible mechanism of action of 6-Gin on A549 tumor were investigated. The major novel findings in the present study were that 6-Gingerol suppresses A549 cells survival and proliferation *in vitro*, and reduced the size of tumor *in vivo*. Further studies implied that 6-Gingerol ameliorated autophagy-dependent ferroptosis by suppressing the expression of USP14, increasing ROS and Fe^2+^ content.

Six- Gingerol has been considered as a potential therapeutic agent due to its inhibitory effects on inflammation, oxidative stress and carcinogenesis (Koch et al., 2017; Alencar et al., 2018). Our study showed that 6-Gingerol treatment could decrease tumor volume, reduce the accumulation of ROS and iron in the tumor, and reduce the expression levels of autophagy and ferroptosis related proteins. We further studied the effect of 6-gingerol on A549 cells *in vitro* and showed that 80 μM of 6-Gingerol was the most effective in inhibiting cell survival and proliferation.

Both tumor-bearing mice and A549 cells displayed a panel of biomarkers based on autophagy and ferroptosis, such as upregulation of Beclin one and LC3II/LC3I, iron overload, lipid peroxidation, inhibition of USP14 activity and induction of the autophagy-related and ferroptosis-related proteins expression, supporting that USP14 is capable of regulating autophagy and ferroptosis. Notably, 6-Gin treatment significantly supressed USP14 expression, indicating that 6-Gin promoted autophagy effects by inhibition of USP14-Beclin 1, which was consistent with previous study (Xu et al., 2016). However, how 6-Gin affects autophagy in tumors involved in rust remains unresolved.

The induction of ferroptosis is an approach to suppressing tumor growth. Ferroptosis has been shown to promote cell death in a variety of cancer cell lines (Hou et al., 2016; Xie et al., 2016). Iron is essential for oxygen transmission, redox reactions and synthesis of metabolites (Ganz and Nemeth, 2012). Normally, iron is stored in the form of transferrin or ferritin. Ferritin is a multimeric protein complex composed of 24 H and L polypeptide subunits that are freely organized in different proportions to form a shell-like nanocage in which Fe^3+^ is stored (Arosio et al., 2009; Bellelli et al., 2016). NCOA4 was identified as a key player in ferritin phagocytosis (Dowdle et al., 2014), which promotes degradation of autophagic ferritin to release Fe^2+^ from ferritin (Mancias et al., 2014). We found that the expression of NCOA4 *in vivo* and *in vitro* was down-regulated and the expression of FTH1 was up-regulated *in vivo* and *in vitro* after treatment with 6-Gingerol (Figures 5A,B,6A,B), which further supported this opinion. GPX4 is an important selenoprotein, which regulates the death of ferroptotic cell by reducing lipid peroxidation (Yang et al., 2014). In our study, the expression of GPX4 decreased significantly, while the ROS content increased. The content of Fe^2+^ was increased, and Fe^2+^ could generate reactive hydroxyl radicals through Fenton chemical reaction, which in turn caused lipid peroxidation and ferroptosis. 6-Gin could effectively up-regulate ferroptosis by inhibiting the expression of USP14. After USP14 overexpression, the A549 cells were more sensitive to ferroptosis with the increased levels of LC3 II/LC3 I.

In summary, this study shows for the first time that 6-Gingerol blocks the proliferation and survival of A549 cells through the autophagy-ferroptosis pathway. The accumulation of iron in cells contributes to the death of A549 cells. These findings suggested that 6-Gingerol could be a potentially useful natural drug against lung cancer.

## Data Availability Statement

The original contributions presented in the study are included in the article, and further queries can be directed to the corresponding authors.

## Ethics Statement

The animal study was reviewed and approved by Animal Experiment Ethics Committee of Nanjing University of Chinese Medicine.

## Author Contributions

SZ and TY designed the study. XC performed the experiments. SZ and TY wrote the manuscript. All authors read and approved the submitted version.

## Funding

The present study was supported by the Priority Academic Program Development of Jiangsu Higher Education Institutions (PAPD)[2018] No. 87 and the Discipline of Chinese Medicine of Nanjing University of Chinese Medicine Supported by the Subject of Academic Priority Discipline of Jiangsu Higher Education Institutions No. ZYX03KF14.

## Conflict of Interest

The authors declare that the research was conducted in the absence of any commercial or financial relationships that could be construed as a potential conflict of interest.

## Glossary

ATF4: activating transcription factor 4
DHE: dihydroethidium
FBS: fetal bovine serum
FTH1: ferritin heavy chain
GFP: green fluorescent protein
GPX4: glutathione peroxidase four
H&E: hematoxylin-eosin
LC3: microtubule-associated protein light chain three
MDA: malondialdehyde
MTT: 3-(4,5-dimethyl-2-thiazolyl)-2,5-diphenyl-2-H-tetrazolium bromide
NCOA4: nuclear receptor coactivator four
ROS: reactive oxygen species
TfR1: transferrin receptor one
USP14: ubiquitin-specific peptidase 14
